# Gene expression analysis of the ovary of hybrid females of *Xenopus laevis *and *X. muelleri*

**DOI:** 10.1186/1471-2148-8-82

**Published:** 2008-03-10

**Authors:** John H Malone, Pawel Michalak

**Affiliations:** 1 Department of Biology, The University of Texas Arlington, Box 19498, Arlington, Texas, 76010, USA; 2 Laboratory of Cellular and Developmental Biology, National Institute of Diabetes, Digestive, and Kidney Diseases, National Institutes of Health, Department of Health and Human Services, Building 50, Room 3341, Bethesda, Maryland, 20892, USA

## Abstract

**Background:**

Interspecific hybrids of frogs of the genus *Xenopus *result in sterile hybrid males and fertile hybrid females. Previous work has demonstrated a dramatic asymmetrical pattern of misexpression in hybrid males compared to the two parental species with relatively few genes misexpressed in comparisons of hybrids and the maternal species (*X. laevis*) and dramatically more genes misexpressed in hybrids compared to the paternal species (*X. muelleri*). In this work, we examine the gene expression pattern in hybrid females of *X. laevis *× *X. muelleri *to determine if this asymmetrical pattern of expression also occurs in hybrid females.

**Results:**

We find a similar pattern of asymmetry in expression compared to males in that there were more genes differentially expressed between hybrids and *X. muelleri *compared to hybrids and *X. laevis*. We also found a dramatic increase in the number of misexpressed genes with hybrid females having about 20 times more genes misexpressed in ovaries compared to testes of hybrid males and therefore the match between phenotype and expression pattern is not supported.

**Conclusion:**

We discuss these intriguing findings in the context of reproductive isolation and suggest that divergence in female expression may be involved in sterility of hybrid males due to the inherent sensitivity of spermatogenesis as defined by the faster male evolution hypothesis for Haldane's rule.

## Background

Frogs of the genus *Xenopus *provide a striking exception to the most widespread generalization in evolutionary biology-Haldane's rule [1-6]. Haldane's rule states that the heterogametic sex (XY or ZW) typically suffers the most dysfunctional effects of interspecific hybridization [7] and the broad applicability of Haldane's rule across diverse groups of organisms suggests that common mechanisms may underlie postzygotic reproductive isolation [5]. *Xenopus *have ZW sex determination and Haldane's rule would predict that hybrid females should suffer the most dramatic effects of hybridization but contrary to expectation, F1 hybrid males are completely sterile and hybrid females are fertile [1,2].

Analyses of spermatogenesis in hybrid males of *X. laevis *× *X. muelleri *have shown that males have a dramatically lower abundance of motile sperm, increased numbers of undifferentiated sperm cells, and larger mature sperm cells compared to parental species [2]. The gene expression pattern for hybrid males shows a striking asymmetric pattern in that relatively few genes are differentially expressed between hybrids and the maternal species (*X. laevis*) whereas there are dramatically more genes differentially expressed between hybrid males and the paternal species, *X. muelleri*. These results suggest intriguing mechanisms operating on the transcriptome in hybrid males of *Xenopus *that may reflect strong maternal and/or species dominance effects [2].

Hybrid females are just as fertile as conspecific species [1] and given the phenotype of hybrid females, a reasonable prediction would be that gene expression should be similar compared to the two parental species. However, given the asymmetrical pattern of expression operating in hybrid males, it is of interest to investigate the pattern of gene expression in hybrid oogenesis, particularly since oogenesis in hybrids does not seem to be affected by the hybrid genetic background compared to hybrid males.

In this study, we analyzed the gene expression pattern of adult ovary in hybrid females of *X. laevis *× *X. muelleri *compared to the two parental species. Our analyses reveal a pattern of asymmetrical gene expression like that in testes of hybrid males but surprisingly there is a dramatic increase in the number of genes misexpressed in hybrid female ovaries compared to the two parental species relative to hybrid males. This increased level of gene misexpression suggests that oogenesis can tolerate dramatically more misexpression compared to spermatogenesis and points further evidence to the sensitive spermatogenesis component of the faster male evolution hypothesis for Haldane's rule.

## Results

**Figure 1:**
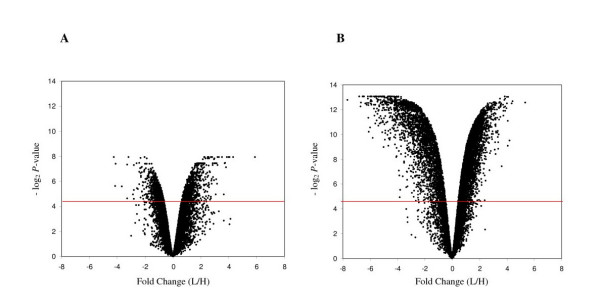
Volcano plots of gene expression. Volcano plots from FDR corrected *t*-tests of statistical significance (vertical axis) against magnitude of expression change (horizontal axis), where each point corresponds to a gene/transcript. Expression change (fold-change) is defined as a log_2_-transformed ratio of mean nonhybrid to mean hybrid expression level. (A) *Xenopus laevis *(L) vs. Hybrids (H); (B) *Xenpous muelleri *(M) vs. Hybrids (H). The red horizontal line denotes FDR adjusted alpha 0.05. The horizontal deviation from 0 towards the right or left reflects hybrid underexpression or overexpression, respectively.

There was a substantial amount of differential expression in hybrid ovary compared to the ovaries of the two parental species. Using adjusted significance tests (*P *< 0.05), about 14% (1,616/11,485) of genes were differentially expressed in hybrid females compared to females of *X. laevis *and 63% (7,279/11,485) of genes were differentially expressed between hybrids and *X. muelleri *(Fig. 1). The number of genes upregulated in hybrids relative to *X. laevis *compared to the number of genes upregulated in *X. laevis *relative to hybrids was the same (839 vs. 777; *G *= 2.38; df = 1; *P *> 0.05) but there were significantly more genes upregulated in *X. muelleri *compared to hybrids (4,349 vs. 2,930; *G *= 139.2; df = 1; *P *< 0.0001). Many of the top 30 most differentially expressed genes for each class of gene expression behavior are expressed sequence tags (ESTs) with little functional information but our results imply that these sequences play a role in oogenesis in *Xenopus*. Of the top 30 candidate genes with known function many have a documented role in oogenesis in other organisms (Table 1, 2, 3, 4). Comparing the two lists of differentially expressed genes showed that about 68% (1105/1616) were common to both *X. laevis *vs. hybrids and *X. muelleri *vs. hybrids. This common set of differentially expressed genes suggests a set of genes that are uniquely expressed in hybrids relative to the two parental species.

**Table 1:** Candidate transcripts upregulated in *X. laevis *compared to hybrids.

ProbeID	GenBankID	Target Gene	Gene Symbol	Description/Molecular Function	Mean Laevis	SD Laevis	Mean Hybrid	SD Hybrid	L-H	adj.P.Val
Xl.1473.1.A1_at	AW147858	EST		Weakly similar to hypothetical protein MGC3731 (H. sapiens)	10.18	0.56	4.28	0.43	5.90	0.0041
Xl.7484.1.A1_at	BJ081331	EST	MGC84382	Intracellular signaling cascade	7.33	0.54	3.01	0.25	4.32	0.0041
Xl.14874.1.A1_at	BM180490	EST		Highly similar to T2D1_HUMAN TRANSCRIPTION INITIATION FACTOR TFIID 250 KD SUBUNIT	7.35	0.35	3.29	0.54	4.06	0.0041
Xl.2557.1.S1_at	BG730898	EST		Weakly similar to A39599 55 K erythrocyte membrane protein – human (H. sapiens)	10.12	0.30	6.10	0.18	4.02	0.0041
Xl.4581.1.A1_at	CB562594	EST	LOC398314	Dihydrolipoamide acetyltransferase	7.34	0.40	3.45	0.37	3.88	0.0041
Xl.14122.1.A1_at	BJ079520	EST	LOC494727		9.51	0.42	5.70	1.02	3.81	0.0072
Xl.12012.2.A1_at	BM191810	EST		Weakly similar to FW1A_HUMAN F-boxWD-repeat protein	7.39	0.34	3.63	0.77	3.76	0.0057
Xl.23885.1.S1_at	BQ388097	EST			7.76	0.50	4.05	0.59	3.71	0.0057
Xl.3628.1.A1_at	BG023013	EST			7.18	1.34	3.52	0.77	3.66	0.0325
Xl.24509.1.A1_at	BJ078115	EST	MGC132176	Heme oxygenase (decyclizing) activity	9.04	0.30	5.50	0.56	3.53	0.0041
Xl.14330.2.S1_at	BG021901	EST	MGC85135	Nucleic acid binding	8.63	0.27	5.34	0.36	3.29	0.0041
Xl.10705.1.A1_at	BJ051966	EST	LOC495457	Weakly similar to A36368 transcription factor CBF, CCAAT-binding – (H. sapiens)	8.67	0.34	5.45	0.79	3.22	0.0064
Xl.1973.1.S1_at	BQ383513	EST	MGC78898	Ubiquitin-protein ligase activity	7.41	0.19	4.19	0.39	3.22	0.0041
Xl.4825.1.A1_at	BG514303	EST			10.16	0.84	6.99	0.62	3.17	0.0182
Xl.14885.1.A1_at	BM180520	EST	MGC82215		8.79	0.46	5.64	0.23	3.15	0.0057
Xl.16512.1.A1_at	BJ050593	EST			7.03	0.48	3.91	1.11	3.12	0.0142
Xl.1448.2.S1_a_at	BG408234	EST		Moderately similar to pallidin; pallid (mouse) homolog, pallidin (H. sapiens)	7.59	0.44	4.50	1.91	3.10	0.0325
Xl.16106.1.A1_at	BG161783	EST	LOC495116		8.24	0.23	5.18	0.69	3.05	0.0057
Xl.25509.1.A1_at	BJ084136	EST	LOC495259		6.09	0.34	3.09	0.23	3.00	0.0044
Xl.25610.1.A1_at	BF025269	EST		Similar to thyroid hormone receptor associated protein 3 (predicted) [Rattus norvegicus]	7.16	0.09	4.19	0.77	2.98	0.0057
Xl.10826.1.A1_at	CB560349	EST	MGC82938	Moderately similar to AAKG_HUMAN 5-AMP-activated protein kinase, (H. sapiens)	8.37	0.31	5.42	0.37	2.95	0.0047
Xl.8907.1.S1_at	AW632884	proliferation-2G4	Pa2g4	Xenopus laevis, Similar to proliferation-associated 2G4	6.95	0.49	4.00	1.03	2.95	0.0148
Xl.6727.1.A1_at	BG234472	EST	LOC780754	Regulation of transcription, DNA-dependent	7.28	0.75	4.33	0.33	2.95	0.0149
Xl.2780.1.S1_at	BQ400343	TIA1	tial1-a	Nucleic acid binding	6.41	0.58	3.52	0.64	2.89	0.0124
Xl.16426.1.A1_at	BJ045456	EST			6.58	0.40	3.71	0.46	2.87	0.0063
Xl.6421.1.A1_at	AW633197	EST	MGC68457		8.68	0.38	5.82	0.21	2.86	0.0057
Xl.22941.1.A1_at	BJ048949	EST	MGC83726	Moderately similar to A57088 nucleoporin-like protein Rab;regulation of GTPase activity	6.79	0.15	3.98	0.43	2.81	0.0041
Xl.25368.2.A1_at	BI444111	EST	MGC69123	Weakly similar to IFR1_HUMAN INTERFERON-RELATED DEVELOPMENTAL REGULATOR 1(H. sapiens)	6.21	0.61	3.42	0.56	2.79	0.0139
Xl.14796.1.A1_at	BM179493	EST	MGC82526		7.10	0.69	4.32	0.68	2.78	0.0182
Xl.19146.3.A1_at	BJ057192	EST	MGC81986		7.88	0.52	5.11	0.90	2.78	0.0151

Top 30 candidate transcripts upregulated in *X. laevis *and differentially expressed between females of *X. laevis *and hybrids. Expression values are in log_2 _scale; SD = standard deviation of expression values. *P *values are adjusted according to FDR moderated t-tests.

**Table 2:** Candidate transcripts upregulated in hybrids compared to *X. laevis*.

ProbeID	GenBankID	Target Gene	GeneSymbol	Description/Molecular Function	Mean Laevis	SD Laevis	Mean Hybrid	SD Hybrid	L-H	adj.P.Val
Xl.4605.1.A1_at	BG552470	EST	MGC83384		3.41	0.39	7.63	0.44	-4.22	0.0041
Xl.19075.1.A1_at	BI675584	EST		Weakly similar to HES2 (Hairy and enhancer of split 2) (H. sapiens)	5.53	1.29	9.67	0.27	-4.14	0.0205
Xl.24502.1.S1_at	BJ098608	EST		Weakly similar to forkhead box P2; (H. sapiens)	4.56	0.70	8.65	0.00	-4.09	0.0060
Xl.23573.1.S1_at	BC041496.1	thymine-DNA glycosylase	TDG	Hydrolase activity, acting on glycosyl bonds	5.55	1.16	9.22	0.15	-3.67	0.0210
Xl.16239.1.A1_at	BJ039322	EST			5.39	1.36	8.66	0.46	-3.27	0.0405
Xl.8630.1.S1_s_at	BC045272.1	MGC53990	MGC53990	Similar to serum-inducible kinase; protein serine/threonine kinase act.	4.74	0.36	8.01	0.75	-3.26	0.0062
Xl.21956.1.S1_at	BC042271.1	MGC53461	MGC53461		8.39	0.22	11.57	0.00	-3.18	0.0041
Xl.24699.1.S1_at	CB984321	EST	MGC79012	Highly similar to alpha cardiac actin (H. sapiens)	3.65	0.36	6.51	1.79	-2.85	0.0327
Xl.10600.1.S1_at	BE491023	EST			4.88	0.94	7.68	0.25	-2.80	0.0256
Xl.11324.1.A1_at	BG552091	EST			3.44	0.46	6.23	0.11	-2.79	0.0064
Xl.13010.1.A1_at	BJ099673	EST			5.43	0.46	8.22	1.89	-2.79	0.0417
Xl.14915.1.A1_at	BM180884	EST			5.11	0.30	7.72	0.35	-2.61	0.0057
Xl.13666.1.A1_x_at	BJ091634	EST			7.61	0.80	10.17	0.37	-2.57	0.0230
Xl.24516.1.S1_at	CB560511	EST	LOC495356	Weakly similar to Apolipoprotein E precursor (H. sapiens); lipid binding	6.41	0.60	8.93	1.00	-2.52	0.0244
Xl.13831.3.S1_at	BJ075928	EST			5.64	0.45	8.05	0.17	-2.41	0.0094
Xl.24058.1.S1_at	BI940804	EST	MGC82121	Highly similar to histone H2A.FZ variant, isoform 1(H. sapiens)	6.99	0.28	9.40	0.09	-2.41	0.0057
Xl.16509.1.A1_at	BJ084267	EST			4.04	0.35	6.44	1.00	-2.40	0.0185
Xl.433.2.S1_at	BC044959.1	neurotrophin receptor B	trkb-b	Protein amino acid phosphorylation	3.68	0.40	6.01	0.00	-2.33	0.0076
Xl.19610.1.A1_at	BJ084191	EST		Similar to Angiopoietin-1 receptor precursor (mTIE2)	4.48	0.05	6.81	0.36	-2.33	0.0043
Xl.75.1.S1_at	D78003.1	c4	c4	Endopeptidase inhibitor activity i fourth component of complement	4.09	0.47	6.41	0.00	-2.32	0.0101
Xl.882.1.S1_at	U07179.1	Ldehydrogenase A	ldha	Oxidoreductase activity	5.29	0.94	7.60	0.06	-2.31	0.0387
Xl.17327.1.A1_at	BI448285	EST	MGC68503		4.77	0.49	7.07	0.19	-2.29	0.0117
Xl.747.1.S1_at	AF170341.1	galectin-1	MGC64502	Sugar binding	5.51	0.21	7.80	0.55	-2.29	0.0075
Xl.545.1.S1_at	AF170344.1	metastasis associated 1	mta2	Transcription factor activity, regulation of transcription	7.85	0.51	10.14	0.13	-2.29	0.0124
Xl.23647.1.S1_at	BC047974.1	cell death 2	pdcd2	Apoptosis	4.11	0.36	6.34	0.40	-2.23	0.0096
Xl.19047.1.A1_at	BI478140	Coatomerprotein	copa	ER to Golgi vesicle-mediated transport i	8.05	0.35	10.28	0.01	-2.23	0.0071
Xl.9113.1.A1_at	BG346438	chimerin	chn1	Signal transduction	6.10	0.76	8.32	0.12	-2.22	0.0276
Xl.16847.1.A1_at	BJ052360	EST			4.22	0.74	6.44	0.36	-2.22	0.0279
Xl.13666.1.A1_at	BJ091634	EST			6.81	0.77	8.97	0.54	-2.16	0.0350
Xl.721.1.S1_at	L09728.1	transcription factor DLL4	Dlx2	Regulation of transcription, DNA-dependent	7.89	0.58	10.04	0.35	-2.15	0.0199

Top 30 candidate transcripts upregulated in hybrids and differentially expressed between females of *X. laevis *and hybrids. Expression values are in log_2 _scale; SD = standard deviation of expression values. *P *values are adjusted according to FDR moderated t-tests.

**Table 3:** Candidate transcripts upregulated in *X. muelleri *compared to hybrids.

Probe ID	GenBank ID	Target Gene	Gene Symbol	Description/Molecular Function	Mean Muell.	SD Muell.	Mean Hybrid	SD Hybrid	M-H	*P *value
Xl.12012.2.A1_at	BM191810	EST		Weakly similar to FW1A_HUMAN F-boxWD-repeat protein 1B (H. sapiens)	8.50	0.22	3.16	0.55	5.35	0.0002
Xl.7034.1.S1_at	BC043865.1	LOC398646	LOC398646	Similar to pantophysin, transporter activity	10.03	0.23	5.60	0.29	4.44	0.0002
Xl.5802.1.A1_x_at	AW764672	EST			9.84	0.32	5.46	0.27	4.38	0.0002
Xl.5299.1.S1_at	BI445766	SEB-4	seb4-a	Nucleic acid binding	8.67	0.44	4.47	1.24	4.19	0.0014
Xl.17322.1.A1_a_at	BJ077543	EST		Weakly similar to myeloidlymphoid or mixed-lineage leukemia 2; ALL1-related gene (H. sapiens)	7.00	0.09	2.90	0.14	4.10	0.0001
Xl.3326.2.S1_a_at	X63427.1	Bmp7	MGC68434	Bone morphogentic protein, ossification, growth factor activity	8.03	0.53	3.94	0.43	4.09	0.0005
Xl.24194.1.S1_at	CD362680	EST	MGC68920	Ribosome biogenesis and assembly	8.36	0.60	4.27	1.21	4.09	0.0019
XlAffx.1.12.S1_at	AF256087.1	Xcat 2	Xcat 2	Xenopus borealis Xcat-2	10.81	0.09	6.79	0.43	4.02	0.0002
Xl.23898.1.A1_x_at	BF428365	EST	MGC82089	Membrane alanyl aminopeptidase activity	8.12	0.12	4.12	0.11	4.00	0.0001
Xl.25735.1.S1_at	BE026658	EST		Weakly similar to GRF1_HUMAN G-rich sequence factor-1 (GRSF-1) (H. sapiens)	9.66	0.23	5.68	0.47	3.97	0.0002
Xl.14298.1.A1_at	BQ383420	EST		Moderately similar to MOB-LAK (Homo sapiens) (H. sapiens)	7.73	0.29	3.82	0.46	3.90	0.0003
Xl.4311.1.A1_at	BM261211	EST			6.75	0.24	2.86	0.37	3.89	0.0002
Xl.25283.1.S1_s_at	BU904283	EST	MGC85348	Highly similar to RL2B_HUMAN 60S ribosomal protein L23a (H. sapiens); structural constituent of ribosome	11.11	0.08	7.22	0.18	3.88	0.0001
Xl.24302.1.A1_at	BG555239	EST			9.69	0.04	5.83	0.05	3.86	0.0001
Xl.61.1.S1_s_at	Y17861.1	LAP2	LAP2	Lamina associated polypeptide 2; nuclear envelope	10.11	0.12	6.28	0.21	3.82	0.0001
Xl.15150.1.A1_at	BJ097608	EST			7.54	0.32	3.75	0.22	3.79	0.0002
Xl.6902.1.A1_at	BM261049	EST	MGC68575	Highly similar to B-cell CLLlymphoma 11A (zinc finger protein); (H. sapiens); nucleic acid binding	7.37	0.47	3.60	0.04	3.76	0.0003
Xl.8049.1.S1_a_at	BC041550.1	Similar to VAMP	MGC53868	Similar to VAMP (vesicle-associated membrane protein)-associated protein A, structural molecule activity	8.22	0.28	4.47	0.43	3.75	0.0003
Xl.6272.1.A1_at	AW782701	MGC83120	MGC83120	Highly similar to Calcium-binding protein p22 (Calcium-binding protein CHP) (H. sapiens); calcium ion binding	10.36	0.23	6.65	0.28	3.71	0.0002
Xl.8805.1.S1_s_at	CB564916	ribosomal protein L4	rpl-4	Ribosomal protein L1; structural constituent of ribosome	11.01	0.19	7.37	1.79	3.64	0.0059
Xl.2546.1.S1_at	CD324865	Psma2	Psma2	Proteasome subunit XC3; ubiquitin-dependent protein catabolism	8.19	0.25	4.58	0.10	3.61	0.0002
Xl.17949.1.S1_at	BG022283	EST	MGC68573	Cytochrome-c oxidase activity	7.41	0.23	3.83	0.21	3.58	0.0002
Xl.25755.1.A1_at	CB756768	EST	LOC734179	Moderately similar to SYQ_HUMAN Glutaminyl-tRNA synthetase(H. sapiens), glutamate-tRNA ligase activity, protein biosynthesis	7.67	0.18	4.09	0.30	3.58	0.0002
Xl.7619.1.S1_a_at	BC045223.1	zf-e326	zf-e326	Intracellular signaling cascade	9.85	0.09	6.28	0.41	3.57	0.0002
Xl.23754.1.S1_at	AW147985	EST	LOC495016		8.87	0.30	5.32	0.01	3.55	0.0002
Xl.23241.1.S1_at	CA988460	EST			8.23	0.37	4.68	0.37	3.55	0.0004
Xl.7661.1.S1_at	BJ097640	EST	LOC495305	Weakly similar to MCA3_HUMAN Multisynthetase complex auxiliary component p18 (H. sapiens)	11.18	0.14	7.64	0.40	3.54	0.0002
Xl.2200.1.A1_at	BM179326	EST		Calcium ion binding	6.64	0.02	3.13	0.11	3.51	0.0001
Xl.1140.1.S1_s_at	X63425.1	Bmp2	Bmp2	Bone morphogenetic protein 2; growth factor activity; ossification	9.29	0.18	5.79	0.07	3.50	0.0002
Xl.15786.1.A1_at	BJ056161	EST	MGC83224	tRNA processing	8.88	0.19	5.40	0.02	3.48	0.0002

Top 30 candidate transcripts upregulated in *X. muelleri *and differentially expressed between females of *X. muelleri *and hybrids. Expression values are in log_2 _scale; SD = standard deviation of expression values. *P *values are adjusted according to FDR moderated t-tests

**Table 4:** Candidate transcripts upregulated in hybrids compared to *X. muelleri*.

ProbeID	GenBank ID	Target Gene	GeneSymbol	Description/Molecular Function	Mean Muell.	SD Muell.	Mean Hybrid	SD Hybrid	M-H	*P *value
Xl.4276.1.S1_at	X53745.1	Cyclin A1	LOC397885	Regulation of progression through cell cycle	5.12	0.58	12.76	0.10	-7.64	0.0001
Xl.8319.1.S1_at	BJ098891	Herz03	Herz03		4.84	0.22	11.65	0.03	-6.80	0.0001
Xl.21809.1.S1_at	BC041555.1	MGC53900	MGC53900	Similar to calcium modulating ligand	3.00	0.31	9.78	0.23	-6.79	0.0001
Xl.17345.1.A1_at	BJ053357	EST	MGC115708	Weakly similar to MIC2_HUMAN T-cell surface glycoprotein E2 precursor (H. sapiens)	3.33	0.21	10.03	0.99	-6.70	0.0002
Xl.4744.1.S1_at	BE491637		LOC495025	Moderately similar to ubiquitin thiolesterase (H. sapiens); ubiquitin-dependent protein catabolism	4.78	0.60	11.43	0.16	-6.65	0.0002
Xl.6585.1.S1_at	BJ080015	Similar to HIV-1 rev binding protein 2	HRB2	RNA binding	3.22	0.20	9.75	0.14	-6.53	0.0001
Xl.21357.2.S1_at	BJ045324	Claudin7L1	MGC53400	Xenopus laevis cldn7L1 mRNA for Claudin7L1, structural molecule activity	3.90	0.27	10.42	0.03	-6.51	0.0001
Xl.14775.1.A1_at	BM179359	EST		Weakly similar to POL2_MOUSE Retrovirus-related POL polyprotein (M. musculus)	3.81	0.18	10.23	0.22	-6.43	0.0001
Xl.3668.1.S1_at	AF450296.1	XLCL2	LOC397879	Xenopus laevis F-box protein (PXP17), meiosis	4.46	0.60	10.82	0.06	-6.36	0.0002
Xl.3401.2.A2_at	BG016692	EST	LOC446970	Similar to axotrophin; likely ortholog of mouse axotrophin (H. sapiens), protein binding	3.72	0.63	9.95	0.14	-6.24	0.0002
Xl.1018.1.A1_at	U44950.1	Vitelline envelope glycoprotein	lzpb-a	Xenopus laevis vitelline envelope 37 k glycoprotein xlZPB	4.10	0.53	10.31	0.29	-6.22	0.0002
Xl.3862.2.S1_x_at	CD361360	Translation factor sui1	gc20	Translation initiation factor activity	3.29	0.41	9.45	0.17	-6.16	0.0001
Xl.7151.1.S1_at	BJ089477	EST	MGC68561	Moderately similar to hypothetical protein FLJ10738 (H. sapiens); 3'-5' exonuclease activity	4.69	0.31	10.84	0.21	-6.15	0.0001
Xl.3536.2.S1_x_at	BF615663	EST	LOC495200	Highly similar to transcription factor BTF3a – (H. sapiens)	4.19	0.26	10.32	1.21	-6.13	0.0004
Xl.23448.1.S1_at	BC041216.1	SWI/SNF	smarce1	Similar to SWISNF, actin dependent regulator of chromatin, regulation of transcription	3.75	0.62	9.88	0.23	-6.13	0.0002
Xl.2060.1.A1_x_at	BJ055271	EST			3.61	0.40	9.69	1.64	-6.08	0.0008
Xl.576.1.S1_at	AF184090.1	fatvg	fatvg		4.94	0.56	11.02	0.03	-6.08	0.0002
Xl.24785.1.S1_at	BM261081	EST	MGC81067		4.41	0.07	10.41	0.03	-5.99	0.0001
Xl.4504.1.A1_at	BJ076394	EST		Weakly similar to ACRC protein; putative nuclear protein (H. sapiens)	4.11	1.10	10.10	0.06	-5.99	0.0007
Xl.7045.1.S1_a_at	BQ398421	EST		Weakly similar to CTF1_HUMAN Cardiotrophin-1 (CT-1) (H. sapiens)	2.67	0.16	8.64	0.24	-5.97	0.0001
Xl.7252.1.S1_at	AY172320.1	Germes	LOC398520		3.60	0.18	9.55	0.27	-5.96	0.0001
Xl.2565.3.S1_x_at	CB561588	Similar to alpha-Tubulin at 84B	MGC53359	Xenopus laevis, Similar to alpha-Tubulin at 84B, microtubule-based movement	4.44	0.41	10.39	0.27	-5.95	0.0002
Xl.7837.1.A1_at	BF232270	EST	MGC132211	Highly similar to hypothetical protein FLJ10900 (H. sapiens), electron transport	2.94	0.43	8.88	0.02	-5.94	0.0001
Xl.25809.1.A1_at	BE026874	EST	MGC80281	Histidine catabolism	4.26	0.49	10.18	0.36	-5.92	0.0002
Xl.6605.1.A1_at	AW632842	EST			3.59	0.26	9.50	0.02	-5.91	0.0001
Xl.4170.2.A1_at	BQ398301	LOC494857	LOC494857	Cell differentiation	4.21	0.59	10.11	0.11	-5.90	0.0002
Xl.1014.1.S1_at	U46131.1	Cdc21 protein	cdc21	Xenopus laevis DNA replication initiator protein, DNA replication initiation, regulation of transcription	4.14	0.46	10.00	0.08	-5.86	0.0002
Xl.2839.1.S1_at	BC041270.1	Protein translocation complex	sec61beta	Similar to protein translocation complex beta	5.91	0.17	11.76	0.08	-5.85	0.0001
Xl.25536.1.A1_at	BE677987	EST		Weakly similar to hypothetical protein MGC2577 (H. sapiens)	4.36	1.14	10.15	0.93	-5.79	0.0012
Xl.14065.1.A1_at	AW147826	EST			3.61	0.15	9.33	0.12	-5.72	0.0001

Top 30 candidate transcripts upregulated in hybrids and differentially expressed between females of *X. muelleri *and hybrids. Expression values are in log_2 _scale; SD = standard deviation of expression values. *P *values are adjusted according to FDR moderated t-tests.

Gene expression between the two parental species was also dramatically different. More than 76% (8,741/11,485) of genes were differentially expressed between females of *X. laevis *and *X. muelleri*. Of these differentially expressed genes, about 60% (5,203/8,741) were upregulated in *X. muelleri *relative to *X. laevis *(5,203 vs. 3,538; *G *= 159.5; df = 1; *P *< 0.0001). Comparing the overlap in genes differentially expressed in the two hybrid contrasts to the three classes of expression behavior between *X. laevis *and *X. muelleri *(*X. laevis *> *X. muelleri; X. laevis *<*X. muelleri*; *X. laevis *= *X. muelleri*) shows a general pattern of semidominance in expression behavior (Table 5). For example, of the 839 genes upregulated in hybrids relative to *X. laevis*; 90% were upregulated in *X. muelleri *relative to *X. laevis*. Similarly, of the 2,930 genes that were upregulated in hybrids relative to *X. muelleri*, 91% were upregulated in *X. laevis *compared to *X. muelleri*. These results suggest a general pattern of intermediate expression in hybrids and are consistent with a semidominant model of expression difference even despite the asymmetrical pattern of misexpression in hybrids compared to the two parental species.

**Table 5:** Overlap of transcripts from comparisons of hybrids and both species.

	L < H	L > H	M < H	M > H
L > M	24	626	2654	24
L < M	753	65	9	3997
L = M	62	86	267	328
Total	839	777	2930	4349

Comparison in the overlap of transcripts recovered as differentially expressed from the two contrasts with hybrids (*Xenopus laevis *(L) vs. hybrids (H) and *X. muelleri *(M) vs. hybrids) and the interspecies contrast (*Xenopus laevis *vs. *X. muelleri*). The congruence between patterns of expression behavior in hybrids compared to the interspecies comparison suggests a model of semidominance where hybrids have an intermediate level of expression compared to the two parental species

## Discussion

Our analysis of hybrid females relative to the two parental species provides key insight into the process of oogenesis in hybrid females and the two parental species. There is an asymmetrical pattern of differential expression with about 4.5 times more genes differentially expressed between hybrids and *Xenopus muelleri *compared to *X. laevis*. This result implies that strong maternal and/or species dominance effects act in oogenesis and these are reflected in the hybrid transcriptome. Hybrid females have a general pattern of semidominance in gene expression with the majority of genes being expressed at intermediate levels compared to the two parental species. Finally, there is a dramatic divergence in gene expression in the ovary between the two parental species with more than 76% of genes differentially expressed between *X. laevis *and *X. muelleri*. This suggests that the process of oogenesis differs widely at the gene expression level between these two species of *Xenopus*.

It is important to consider the methodology used to gather the samples of RNA for this study. Samples of ovary (50 mg portions) were dissected and then homogenized in RNA extraction solution. Therefore, we gathered a sample of ovary rather than the entire ovary and this sample is a heterogeneous representation of oogenesis, rather than a direct assessment of specific stages of oocyte development. Given the heterogeneous nature of the tissue used to gather RNA, it is even more surprising that we found such strong effects. Increased heterogeneity among samples would decrease the ability to reject the null hypothesis that gene expression for a particular gene is the same between hybrids and conspecifics. Increased heterogeneity among samples would increase the standard error and thereby decrease power to reject the null hypothesis. In fact though, even despite the heterogeneous nature of the samples collected, we still reject a large portion of null hypotheses suggesting that our microarray results represent real biological effects, rather than statistical artifacts. Additionally, our results remain robust even when using different normalization techniques (scaling and Robust Multichip Averaging) providing further confidence that our results are not statistical artifacts (not shown).

The top 30 candidate genes for the contrasts between hybrids and the two parental species provide many genes with known roles in mitosis, meiosis, and oogenesis in general (Table 1, 2, 3, 4). One EST, *MGC132176*, is predicted to have heme oxygenase activity and this EST was upregulated 12 times in *X. laevis *relative to hybrids. Heme oxygenase plays a role in regulating ovarian steroidgenesis in rats and our results suggest this may be the case in *Xenopus *[8] as well. Of the genes with known function, many have been documented to play a role in oogenesis. For example, the proliferation associated protein *PA2G4*, which was upregulated in *X. laevis *about 8 times higher than in hybrids, has been previously isolated from *Xenopus *oocytes and is believed to play an important role in DNA replication and cell cycle progression [9]. One EST that is similar to the human transcription factor *Hairy and enhancer of split 2*, was upregulated 18 times in hybrids relative to *X. laevis*, and is known to be regulated by reproductive hormones in adult rat ovary [10]. Neurotrophin receptor B (*Trkb-b*) was upregulated 5 times in hybrids relative to *X. laevis *and plays a critical role in ovulation, steroid secretion, and follicular development in the ovary of rodents and humans [11-15].

Examining candidate gene lists for the *Xenopus muelleri *vs. hybrid comparison also reveal many genes involved in oogenesis. For example two bone morphogenetic proteins, *Bmp7 *and *Bmp2*, are 17 and 11 times respectively upregulated in *X. muelleri *relative to hybrid females. Bone morphogenetic proteins are part of a class of proteins involved in the development and patterning of the adult ovary [16,17]. *Xcat-2 *was upregulated 17 times more in *X. muelleri *relative to hybrids and is involved in the formation of germ plasm during stage I oocytes of *Xenopus *[18,19]. Another gene of interest is *LAP2*, upregulated 14 times higher in *X. muelleri *compared to hybrids and specific isoforms of *LAP2 *are expressed exclusively in the ovary of anurans and salamanders [20-22]. *Cyclin A1*, the most divergently expressed gene, was upregulated nearly 200 times higher in hybrids relative to *X. muelleri *and expressed the same in *X. laevis *and hybrids. *Cyclin A1 *plays a major role in mammalian gametogenesis and meiosis [23] and is a partner of *Cdk2*, a key gene involved in the cell cycle both in mitosis and meiosis. Female and male knockout *Cdk2 *mice are viable but both infertile [24]. Curiously, disruption of *Cyclin A1 *expression results in male infertility but not female infertility and specifically causes the developmental arrest of spermatogenesis during meiosis I [25,26]. A vitelline envelope glycoprotein, *lzpb-a*, was upregulated 75 times in hybrids relative to *X. muelleri*, and vitelline envelope proteins have an obvious role in the formation of oocytes during amphibian oogenesis [27]. Finally, *Germes*, a gene that localizes to the germ plasm during early oogenesis in *Xenopus *[28] was upregulated 62 times higher in hybrids compared to *X. muelleri*.

Perhaps the most surprising result of our analyses is that hybrid females are fertile yet have a dramatic increase in gene misexpression compared to hybrid males which are completely sterile [2]. These results would seem to contradict what we might intuitively predict; specifically that it seems reasonable to assume that normal phenotypes should have greater similarity in expression profiles and perturbed phenotypes should have greater divergence in expression. Hybrid males, which are completely sterile, have only 56 genes misexpressed in testes compared to both parental species whereas hybrid females, which are fertile, have nearly 20 times more genes misexpressed (1,105) in ovaries. However, these results are consistent with patterns of sex-biased gene expression in which female-biased genes were found to be more divergently expressed between species compared to male-biased genes [29] providing further evidence that this pattern represents real biological effects. Thus, we are left with a question, how can the process of oogenesis tolerate such dramatic differences in the level of gene expression, whereas the process of spermatogenesis in hybrid males has relatively few genes misexpressed yet results in complete sterility.

To date, there has been little exploration of this question because studies of gene expression and reproductive isolation have focused on the sterility phenotype which typically involves males. However hybrid females of *Drosophila melanogaster *and *D. simulans*, which are sterile, have been analyzed and hybrids had a majority of genes misexpressed compared to the two species [30]. Recent work has shown that critical genes involved in mammalian female reproduction undergo rapid diversification due to retrotransposed genes in *Mus musculus *[31] and these results may provide a clue to the divergent expression pattern occurring in females of *Xenopus *which could be a general pattern of female reproduction.

*Xenopus *do not conform to a fundamental generalization in evolutionary biology-Haldane's rule [1-7]. Patterns of sex-biased gene expression and comparisons between taxa in which the sex chromosome constitution is reversed suggest that the sensitive spermatogenesis component of the faster-male evolution hypothesis [3,32] is the best explanation for sterile males in *Xenopus *even though females are the heterogametic sex. Given the divergent pattern of expression in hybrids and females between species, we suggest the following scenario to explain hybrid male sterility in *Xenopus*.

First, oogenesis relies on a staggering amount of gene expression with up to 45% of all mouse genes and 55% of all *Drosophila *genes expressed in the mature oocyte [31,33] and additionally this abundant transcription results in maternally deposited RNAs and proteins which foster oocyte growth and early development [31,34]. In particular, much of this RNA deposition functions to localize coding and non-coding RNAs essential to germ cell development into a distinct subcellular domain that can be moved into the vegetal cortex of the oocyte. Interestingly, RNAs localized in the germ plasm may not be translated for years highlighting the importance of the germ plasm as a storage unit for RNA and furthermore many of these stored RNAs are involved in translational regulation of germ cell specific expression [35-38].

We find dramatic differences in gene expression between females of two species of *Xenopus *and many of the most dramatic differences have to do with Early/METRO pathway (e.g. *Germes*, *Fatvg*, *Cyclin A1*) of germ plasm specification [38]. These dramatic, sometimes 200 times different, RNA abundance levels indicate a major difference in the amount of key genes involved in germ plasm specification and maternally loaded RNAs between species. This result would suggest that each species has a divergent way of completing oogenesis with regard to gene expression.

During fertilization, sperm fertilize an egg and this starts the dramatic changes that turn the mature oocyte into a functioning zygote [39]. Each sperm delivers a haploid paternal genome along with mature RNA that initiates and directs subsequent development [40]. The interaction between the paternal genome and stored maternal RNA must coevolve in such a way to ensure successful development. Consider now sperm from a different species, adapted for fertilizing eggs of its conspecific species, that now successfully fertilizes an egg from a different species. As our data suggest, the paternal genome will now interact with a radically different embryo with drastically different amounts of stored maternal RNAs. We therefore suggest the possibility that disruption of spermatogenesis in adult hybrid males occurs because of radically divergent expression in females during oogenesis. Oocytes armed with pools of RNA adapted for one species, work in the sense that they can be fertilized and develop but, the initial differences in maternally stored RNAs generate subsequent dysfunctions in males because molecular interactions that generate the adult testis and subsequent spermatogenesis are misregulated due to the differences in maternally stored RNA populations. Spermatogenesis is special in the sense that during early development key factors fail to interact properly to generate a normally functioning testis.

Several genes from our microarray results suggest directions by which this hypothesis could be tested. One example is *Cyclin A1 *which was upregulated about 200 times more in hybrids and 170 times more in *X. laevis *compared to *X. muelleri*. Knocking out *Cyclin A1 *in mice causes completely sterility and the interaction between *Cyclin A1 *and *cdk2 *is crucial for normal development [24,25]. *Cyclin A1 *is also known to be maternally deposited in *Xenopus *and regulates the progression of the cell cycle and apoptosis [41]. Our hypothesis suggests that factors like *Cyclin A1 *which are loaded into embryos in drastically different amounts may play a role in misdirecting the development of the hybrid testis.

## Conclusion

Our work provides an important first glimpse into the expression pattern of hybrid females and parental species. We find an asymmetrical expression pattern similar to the pattern of expression in hybrid male *Xenopus *and allotetraploid *Arabidopsis *[2,42]. However, hybrid females have a dramatic increase in the number of misexpressed genes compared to sterile males and we suggest that this gene expression divergence plays a role in hybrid male sterility. Our results call for attention as to how divergent expression in females plays a role in reproductive isolation between species.

## Methods

### Microarray Experiments

RNA was extracted from adult ovary in *Xenopus laevis *(n = 4), hybrids of *X. laevis *× *X. muelleri *(n = 2) and *X. muelleri *(n = 3). Hybrid individuals were produced by crossing maternal *X. laevis *with paternal *X. muelleri*. Origin of parents and methodology for creating hybrids has been described elsewhere [2,29]. Sufficient numbers of normal hybrid females from the reciprocal cross were unable to be produced because the reciprocal cross produces increased mortality and the offspring that survive have a high proportion of limb abnormalities [1]. Individual adults were euthanized with MS-222 and 50 mg of ovary was dissected and homogenized in RNA extraction solution using a hand held pestle. RNA was recovered using GeneHunter and Ambion RiboPure total RNA kits. Samples of RNA were checked for purity by examination of the 28S and 18S ribosomal RNA bands from denaturing gel electrophoresis, by 260/280 ratios from scans with a Nanodrop ND 1000 spectrophotometer, and by readouts of the Agilent Bioanalyzer. Total RNA samples were prepared and hybridized to Affymetrix *Xenopus laevis *GeneChip Genome Arrays at the University of Texas Southwestern Medical Center Microarray Array Core Facility following standard Affymetrix protocols. Affymetrix Microarray Analysis Suite (MAS) v.5.0 was used to scan and process each microarray chip. The signals of quality control and poly(A) transcripts revealed that hybridizations were of high quality in all chips. Quality control probe sets (i.e., spike in and housekeeping genes) were removed in subsequent statistical analyses. Hybridizing RNA from a heterospecific species to a microarray designed for a related species can have a dramatic impact on the signal recovered from microarrays [43-46]. To control for this effect, we used an electronic mask generated from hybridizing genomic DNA from *X. laevis *and *X. muelleri *onto the *X. laevis *microarray [2]. This mask which screens out probes that have significant sequence divergence in *X. muelleri *provides 11,485 probesets/genes for further analysis.

### Data Analysis

We conducted three separate comparisons to uncover patterns of differential expression between *Xenopus laevis *and hybrids, *X. muelleri *compared to hybrids, and *X. laevis *compared to *X. muelleri*. First, the *Xenopus laevis *and hybrid chips were normalized using Robust Multichip Averaging (RMA) express software [47] using default parameters for background correction and quantile normalization. These RMA normalized data were then imported into the R statistical environment and tested for differences in expression between *X. laevis *and hybrids for each of the 11,485 genes using a moderated *t*-statistic based on an empirical Bayes method in the Limma package found in Bioconductor [48]. The TopTable function was then used to output the False Discovery Rate (FDR)-adjusted *P*-values and we considered genes with adjusted *P*-values less than 0.05 to be differentially expressed. Goodness of fit tests (*G*), based on the difference between the observed and the expected (under the null hypothesis of equal class probabilities) number of genes, were performed to test whether there was enrichment in the number of genes up-regulated in particular comparisons [49]. We normalized *X. muelleri *and hybrid chips together using RMA and repeated the analyses to uncover differential expression between *X. muelleri *and hybrids. Finally, we normalized *X. laevis *and *X. muelleri *chips together using RMA and repeated the analysis to uncover genes misexpressed between the two species. Separate normalizations were performed for each comparison in keeping with the assumptions of RMA normalization.

## Authors' contributions

JHM conceived of the study, collected the data, performed the statistical analyses, and wrote the manuscript. PM participated in its design and coordination and edited the manuscript. All authors read and approved the final manuscript.
